# Type-1 Seronegative Autoimmune Pancreatitis: A Rare Case of Autoimmune Pancreatitis with Sclerosing Cholangitis and Hashimoto’s Disease

**DOI:** 10.7759/cureus.2624

**Published:** 2018-05-14

**Authors:** Muhammad Tahir, Amanpal Singh

**Affiliations:** 1 Internal Medicine, Jacobs School of Medicine and Biomedical Sciences, University at Buffalo, State University of New York, Buffalo, New York; 2 Gastroenterology, Mercy Medical Group. California.

**Keywords:** autoimmune pancreatitis, primary sclerosing cholangitis, hashimoto, aip

## Abstract

Autoimmune pancreatitis (AIP) is very rarely reported in the literature. It is one of the immunoglobulin-G (IgG) related diseases that commonly presents with abdominal pain, mass, jaundice, and weight changes. The disease also has extrapancreatic manifestations, of which the most common is autoimmune sclerosing cholangitis. We report a case of autoimmune pancreatitis that was further found to be associated with Hashimoto's thyroiditis and sclerosing cholangitis. The clinical manifestations vary and it is important to exclude pancreatic malignancy before diagnosing any patient with AIP. Although further studies need to be done, currently the treatment of choice is steroid therapy. Physicians should also screen patients for other autoimmune diseases to rule out any concern.

## Introduction

Seronegative pancreatitis is a rare disease and relates to one of the immunoglobulin-G (IgG) diseases. The common presentation is abdominal pain, mass, jaundice, and weight changes. The clinical manifestations are variable and the concern for malignancy should be ruled out. We report a case of autoimmune pancreatitis associated with Hashimoto's thyroiditis. This case is a very unique regarding its comorbid pattern and presentation.

## Case presentation

**Figure 1:**
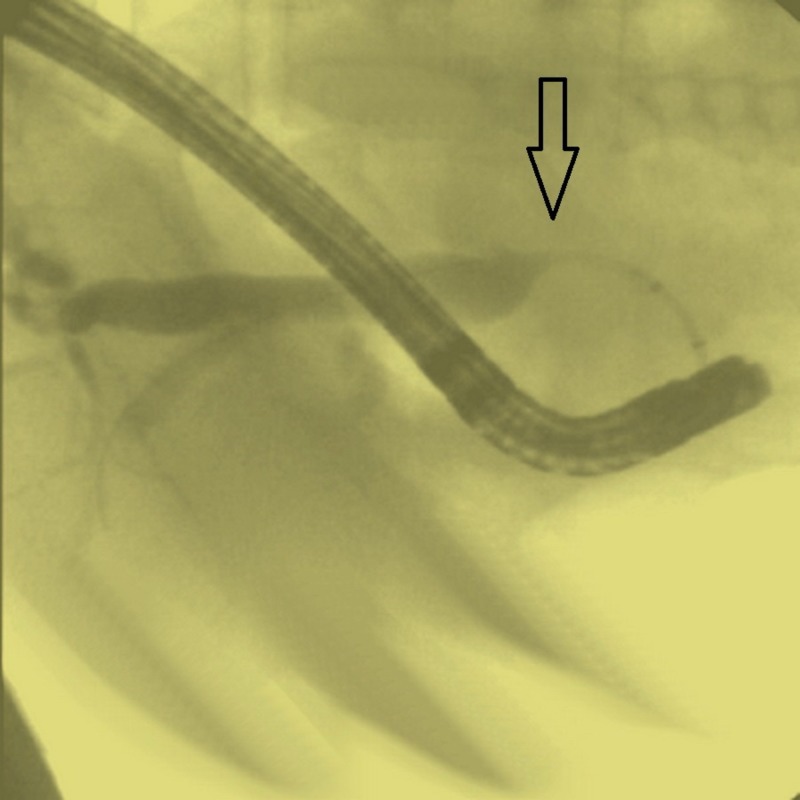
Endoscopic retrograde cholangiopancreatography (ERCP) showing distal stricture in the common bile duct (CBD).

A 41-year-old female with no past medical history presented with acute onset of abdominal pain that was associated with weight loss and painless jaundice. She was stabilized in the emergency room and was admitted for further workup. The physical examination was unremarkable except scleral icterus. The lab workup showed liver enzymes dysfunction (alanine transaminase 144 U/L, aspartate aminotransferase 122 U/L, and alkaline phosphatase 331 IU/L) with conjugated hyperbilirubinemia (5.4 mg/dl). The screen for antinuclear antibody, antimitochondrial antibody, and anti-smooth muscle antibody was negative. There was a marginal increase in total protein to 9 g/dl and an immunoglobulins assay was performed. It showed an increase in IgG total, i.e., 15.1 g/dl, and the IgG subclass analysis showed an increase in IgG-4 level, i.e., 155 mg/dl. The tumor marker screen was negative revealing normal level of cancer antigen 19-9. Imaging showed lymph node enlargement close to celiac plexus origin along with minimal calcification of the pancreatic head. It also revealed the dilatation of the biliary tree. A gastroenterology team was consulted and endoscopic retrograde cholangiopancreatography (ERCP) was performed. The ERCP showed distal stricture in the common bile duct (CBD), which was relieved with stenting (Figure 1).

The histopathology from ERCP brushing was suggestive of primary sclerosing cholangitis (PSC). The endoscopic ultrasound (EUS) was unremarkable. After exclusion of other differentials with negative imaging and tumor marker screen, the diagnosis of autoimmune pancreatitis was made. The patient was treated with steroid therapy and improvement was noticed regarding the subjective and objective aspect. Interestingly, further screening to rule out autoimmune concern revealed the patient to be hypothyroid with Hashimoto profile (increased anti-thyroid peroxidase antibodies), for which she was also started on thyroxine treatment. The patient was followed further for four to six months and she has been doing fine with no concern regarding her medical problems.

## Discussion

Although the topic of AIP is being described worldwide, most of the early literature pertaining to AIP came from Japan perhaps due to increased recognition [1]. AIP has been associated with other autoimmune disorders as well, as in our patient who had Hashimoto's thyroiditis along with PSC. There are two types of AIP mentioned in the literature. Type-1 AIP is involved as one part of the systemic IgG4-positive disease whereas the Type-2 AIP is a non-IgG-4 disease and without any systemic manifestation [2]. The clinical manifestations vary and occur in the pancreas, biliary tract, and other organs. The literature described few most common presentations including mass-like symptoms, recurrent pancreatitis, duct strictures and peripancreatic vascular complications.

The diagnosis should be especially considered in those who have an autoimmune disease and present with nonspecific abdominal symptoms. It should always be distinguished from pancreatic cancer. As per the Mayo Clinic HISORt criteria *(histology, imaging, serology, other organ involvement and response to therapy),* a biopsy is usually required to make the diagnosis [3]. EUS imaging is also low sensitive, i.e., 43% [4]. For any biliary pathology, ERCP should be done to address the concerns, etc. Biochemical testing includes IgG level, which is an important part of the workup if you are diagnosing someone with AIP. Using a cutoff of 135 mg/dL, the sensitivity and specificity of serum IgG4 for distinguishing AIP from pancreatic cancer were 95% and 97%, respectively, although it has been reported lower lately in literature. International Consensus Diagnostic Criteria (ICDC) guidelines are also very important and helpful for diagnostic purposes.

Regarding the treatment, there has been no randomized clinical trial and most of the data has been from observational studies. AIP responds to steroid therapy for two to four weeks with prednisolone [5]. The relapses have also been reported [6]. These patients can be managed with a repeat course and/or maintenance therapy with a goal of lowest possible steroid dose to help keep them in remission.

## Conclusions

Given the variable clinical presentation and symptoms overlap, a thorough evaluation by multidisciplinary team members should be encouraged to rule out related concerning pathologies, and every patient should be screened for autoimmune diseases.
